# Phase 1 studies of the safety and immunogenicity of electroporated HER2/CEA DNA vaccine followed by adenoviral boost immunization in patients with solid tumors

**DOI:** 10.1186/1479-5876-11-62

**Published:** 2013-03-08

**Authors:** Claudia Marcela Diaz, Alberto Chiappori, Luigi Aurisicchio, Ansuman Bagchi, Jason Clark, Sheri Dubey, Arthur Fridman, Jesus C Fabregas, John Marshall, Elisa Scarselli, Nicola La Monica, Gennaro Ciliberto, Alberto J Montero

**Affiliations:** 1 Medical University of South Carolina, Hollings Cancer Center, Charleston, USA; 2 H. Lee Moffitt Cancer Center, Tampa, USA; 3 IRBM "P. Angeletti" - MSD, Pomezia - Rome, Italy; 4 Merck Sharp & Dohme Corp, Whitehouse Station, NJ, USA; 5 University of Miami, Sylvester Comprehensive Cancer Center, Miami, FL, USA; 6 Lombardi Cancer Center, Georgetown University, Washington DC, USA; 7 Takis, S.r.l., Rome, Italy; 8 Incyte Corporation, Wilmington, USA; 9 Okairos, S.r.l., Rome, Italy; 10 Idera Pharmaceuticals Inc., 167 Sidney Street, Cambridge, MA 02139, USA; 11 National Cancer Institute, Fondazione G. Pascale, Napoli, Italy

**Keywords:** DNA vaccine, Adenoviral vaccine, Electroporation, Prime-boost, Solid tumors, Cell-mediated immune response

## Abstract

**Background:**

DNA electroporation has been demonstrated in preclinical models to be a promising strategy to improve cancer immunity, especially when combined with other genetic vaccines in heterologous prime-boost protocols. We report the results of 2 multicenter phase 1 trials involving adult cancer patients (n=33) with stage II-IV disease.

**Methods:**

Patients were vaccinated with V930 alone, a DNA vaccine containing equal amounts of plasmids expressing the extracellular and trans-membrane domains of human HER2, and a plasmid expressing CEA fused to the B subunit of *Escherichia coli* heat labile toxin (Study 1), or a heterologous prime-boost vaccination approach with V930 followed by V932, a dicistronic adenovirus subtype-6 viral vector vaccine coding for the same antigens (Study 2).

**Results:**

The use of the V930 vaccination with electroporation alone or in combination with V932 was well-tolerated without any serious adverse events. In both studies, the most common vaccine-related side effects were injection site reactions and arthralgias. No measurable cell-mediated immune response (CMI) to CEA or HER2 was detected in patients by ELISPOT; however, a significant increase of both cell-mediated immunity and antibody titer against the bacterial heat labile toxin were observed upon vaccination.

**Conclusion:**

V930 vaccination alone or in combination with V932 was well tolerated without any vaccine-related serious adverse effects, and was able to induce measurable immune responses against bacterial antigen. However, the prime-boost strategy did not appear to augment any detectable CMI responses against either CEA or HER2.

**Trial registration:**

Study 1 – ClinicalTrials.gov, NCT00250419; Study 2 – ClinicalTrials.gov, NCT00647114.

## Background

Interest in cancer immunotherapy has been revived with the 2010 US Food and Drug Administration approval of sipuleucel-T, the first approved therapeutic vaccine for the treatment of advanced cancer [1,2]. The recent approval of the CTLA-4 monoclonal antibody ipilimumab has generated further interest in immune-based therapies in cancer [3,4]. However, while cancer vaccinations are well-tolerated, the vast majority of peptide/protein and cell-based vaccines have failed to induce sufficient immune responses to provide long-lasting clinical benefits [5]. DNA plasmid-based vaccines have significant advantages over cell-based or peptide platforms—they are highly amenable to modifications (e.g., multiple epitopes, codon optimization, inclusion of danger signals, and/or cytokines) that could result in enhanced immunogenicity and superior clinical activity [6,7].

Although the use of DNA vaccines has shown great promise in preclinical models, results of early phase clinical trials have been rather disappointing [8]. Challenges for DNA vaccines include the fact that the amount of plasmid DNA that can be injected in humans is substantially lower than in preclinical studies, and the poor level of cellular DNA uptake. However, DNA injection into skeletal muscle followed by a short electrical stimulation, also referred to as electro-gene-transfer or electroporation (EP), significantly enhances DNA uptake and gene expression [9,10]. In the case of self-antigens (e.g., tumor-associated antigens), this approach has been shown, in some preclinical studies, to result in the induction of strongly protective immune responses [11]. Although the mechanism remains unknown, it is speculated that transient pores on the cell surface could lead to enhanced antigen expression and transient tissue damage may lead to the recruitment of inflammatory cells and production of cytokines [12].

Replication-defective E1-deleted recombinant adenoviruses (Ad) have proven to be very efficacious in inducing strong antibody and cellular antigen-specific immune responses against a variety of antigens in several species,[13-17] and have been tested in human clinical trials with antigens from HIV-1 [18,19]. Adenoviral vectors are also being evaluated in clinical trials using DNA vaccine priming regimens followed by Ad vector–boosted immunizations (heterologous prime-boost immunization regimens) [20]. Results indicate that these regimens are capable of generating higher amplitude and more durable immune responses, leading to potentially better prophylactic and therapeutic efficacy in a variety of preclinical disease models [21-23]. The combined treatment of Ad vectors with DNA electroporation (DNA-EP) may give rise to superior immune responses that could result in clinical benefit in cancer patients [6,24].

Because many solid tumors overexpress human epidermal growth factor receptor 2 (HER2) and carcinoembryonic antigen (CEA), they are good targets for immunotherapy [25,26]. Both HER2 and CEA are cell surface markers involved in cell-mediated immunity (CMI) and antibody-dependent cell-mediated cytotoxicity (ADCC) [25,27]. In a series of preclinical studies, we have shown that when inserted into Ad vectors or delivered via DNA-EP, codon-optimized versions of the CEA and HER2 complementary DNAs (cDNAs) are capable of inducing potent T- and B-cell immune responses and break tolerance to self in mice and nonhuman primates [13,28,29]. Immune responses are further enhanced when CEA is fused to the B subunit of *Escherichia coli* heat labile enterotoxin (LTB),[13,30] and when HER2 is truncated to exclude the intracellular domain [31]. Furthermore, the heterologous DNA-EP prime-Ad boost vaccination regimens have potent antitumor efficacy in colon and breast cancer mouse models when animals were vaccinated against CEA or HER2, respectively [13,32]. Based on these results, we generated a dual-component human vaccine V930 DNA-EP/ V932 Ad. V930 is a bivalent DNA plasmid vaccine consisting of 2 separate plasmids—one expressing the extracellular (ECD) and transmembrane (TM) domains of human HER2, and the other expressing human CEA fused to the LTB. V932 is a dicistronic adenoviral vaccine vector, which encodes both human CEA fused to LTB and the truncated version of human HER2 tumor antigen (HER2-ECDTM). CEA was fused to LTB with the intent to enhance immune response to CEA by enhancement of cross-priming. Expression of CEA-LTB is driven by the human cytomegalovirus immediate early (CMV IE) promoter, whereas the mouse CMV IE promoter drives expression of HER2-ECDTM. Since preclinical and clinical data have shown that DNA vaccines appear to be effective at priming when followed by viral vector boosting, the combined treatment with DNA-EP and adenoviral vaccine may give rise to superior immune responses that may result in increased efficacy. We conducted 2 separate phase 1 trials in cancer patients whose tumors expressed CEA and/or HER2 in order to evaluate the safety/tolerability, as well as the immunogenicity, of the bivalent DNA plasmid vaccine V930 with EP injection alone (Study 1) or as a heterologous prime-boost approach involving V930 DNA-EP first, followed by V932 Ad (Study 2).

## Methods

### Study designs

**Figure 1:**
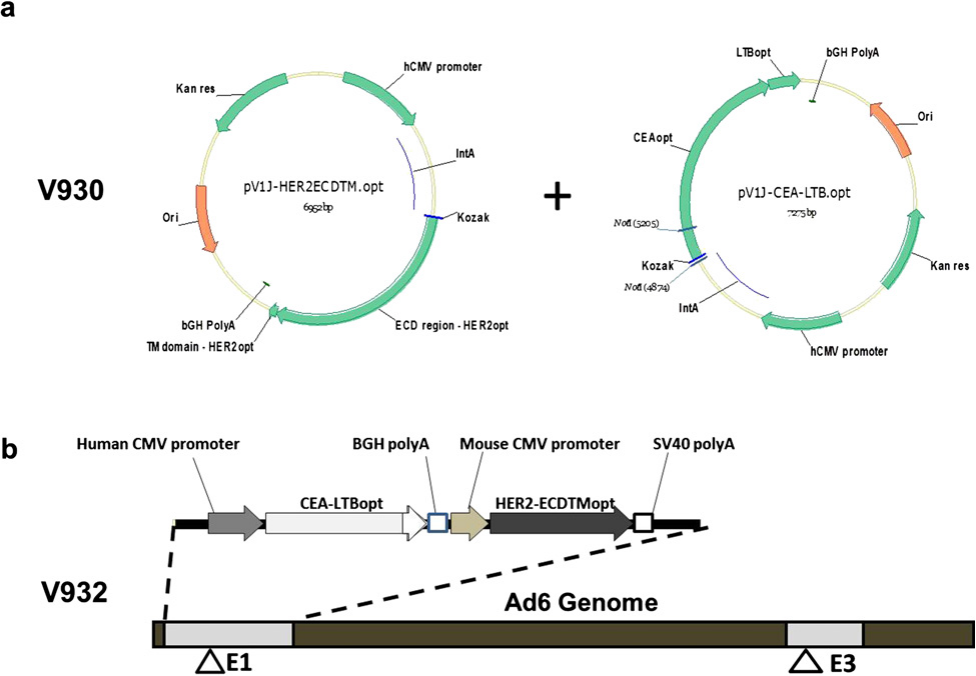
**V930 DNA plasmids (a) and V932 adenoviral vector (b) encoding for HER2/neu and CEA.** V930 is a bivalent DNA plasmid vaccine consisting of a plasmid expressing the ECD and TM domains of HER2 and a plasmid expressing CEA fused to the B subunit of *E coli* LTB. V932 Ad encodes human CEA fused to LTB and the truncated version of human HER2 tumor antigen (HER2-ECDTM). The CEA-LTB expression is driven by the human CMV IE promoter, whereas mouse CMV IE promoter drives the expression of HER2-ECDTM.

Two multicenter, phase 1, open-label dose escalation trials were conducted in adult cancer patients with histologically confirmed stage II-IV solid malignancies expressing HER2 and/or CEA. The phase I trials were designed with only a low dose and a high dose cohort, with escalation to the high dose being done after 6 patients completed vaccinations without any severe adverse toxicities. The primary end point of Study 1 (ClinicalTrials.gov identifier: NCT00250419; http://clinicaltrials.gov/ct2/show/NCT00250419; Protocol 002) was to determine the safety and immunogenicity of escalating doses of V930 administered as an intramuscular (IM) vaccination followed by EP. The primary end point of Study 2 (ClinicalTrials.gov identifier: NCT00647114; http://clinicaltrials.gov/ct2/show/NCT00647114; Protocol 003) was to assess the safety/tolerability and immunogenicity of the heterologous vaccine prime-boost approach consisting of V930 DNA-EP at a fixed dose followed 4 and 6 weeks later by vaccination with V932 Ad, a dicistronic adenovirus subtype 6 viral vector vaccine coding for both CEA and HER2 (Figure 1). In both studies, gene delivery into cells was aided by EP with the MedPulser™ DDS immediately following intramuscular injection of V930 DNA.

### Study participants

Both studies were conducted in accordance with principles of the Declaration of Helsinki, in compliance with Good Clinical Practice (GCP), and approved by the appropriate institutional review boards and regulatory agencies. A written informed consent was obtained from patients prior to participating in the studies, in accordance with GCP. Men or women with stage II-IV solid malignancies shown to express HER2 and/or CEA by immunohistochemistry, at least 18 years old at the time of clinical trial entry were enrolled. Patients were required to have completed standard adjuvant therapy (radiotherapy, chemotherapy, or biologic therapy) at least 1 month prior to enrollment or refused standard adjuvant therapy when rendered disease-free following surgery. Additionally, for locally advanced or metastatic cancers, the patient’s disease status, assessed within 2 weeks prior to enrollment, had to have been stable (≥3 months). Additionally, patients were required to have a Karnofsky performance status of 80 to 100 at the time of study entry. Women of childbearing potential had to demonstrate a non-gravid state prior to and had to agree to contraceptive use or abstinence during the study period.

Primary exclusion criteria included known history of HIV or hepatitis B or C; active medical conditions (e.g., arrhythmia or myocardial infarction) within the last 3 months; presence of an implantable cardiofibrillator and/or pacemaker; active psychiatric or substance abuse disorder; history of splenectomy or autoimmune disorders; receiving immunosuppressive therapy; known history of coagulopathy or thrombocytopenia prohibiting IM injections; symptomatic ascites or pleural effusion; recent receipt of a non-study vaccine or any investigational drug; or allergy to any of the vaccine components. Patients with a history of a second malignancy were also excluded, with some exceptions.

### Vaccination schedule

**Figure 2:**
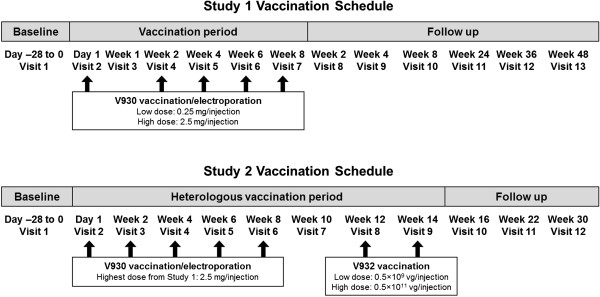
**Vaccination schedule with V930 DNA-EP alone (Study 1) and combined V930 DNA-EP→ V932Ad (Study 2). **Patients who safely tolerated the highest dose of V930 DNA-EP (2.5 mg/injection) in **Study 1 **were allowed to enroll directly into **Study 2**, provided they had completed all 5 V930 vaccinations at least 4 weeks and no more than 24 weeks prior to entry and met all other eligibility criteria.

Patients enrolled in Study 1 were vaccinated with V930 DNA-EP at one of 2 different sequential dose levels: 0.25 mg DNA/injection (low dose) or 2.5 mg DNA/injection (high dose). At each dose level, patients received a series of 5 IM injections of V930 DNA-EP administered every 14 days (on days 1, 15, 29, 43, and 57) as a 0.5-mL injection given at a 90° angle into the deltoid muscle of alternating arms using a 1.0-mL syringe with a 27-gauge, 1.27-cm needle (Figure 2). Within 2 minutes of the injection, each patient was given an EP IM injection consisting of two 60-millisecond pulses using the MedPulser™ DDS device.

In Study 2, patients also received a series of 5 V930 DNA-EP vaccinations (2.5 mg/injection, the highest dose evaluated in Study 1) administered the same way as in Study 1. This was followed by a prime-boost approach, with 2 series of V932 Ad injections given 4 and 6 weeks after the fifth vaccination with V930 DNA-EP (Figure 2). Patients went on to receive one of 2 possible dose levels of V932 Ad: 0.5 × 10^9^ vg/injection (low dose) or 0.5 × 10^11^ vg/injection (high dose). Provided they continued to meet eligibility criteria, patients from Study 1 who had completed the high-dose regimen of V930 DNA-EP (2.5 mg/injection) were eligible to enroll directly into Study 2, as long as at least 4 weeks but no more than 24 weeks had elapsed since the fifth and final injection of V930 DNA-EP. Patients were followed for 1 year after the last vaccination for safety and immunogenicity.

Patients only received the respective high-dose level of either V930 DNA-EP (Study 1) or V932 Ad (Study 2) after 6 patients had completed the entire low-dose vaccination regimen of each study and a 4-week post-observation period. No intra-patient escalation was allowed in either study.

### Study procedures

In both studies, patients were observed for approximately 30 minutes immediately after each treatment for adverse reactions. Patients were asked to complete a treatment report card to record oral evening temperatures, and any injection-site reactions for 5 days following each treatment, as well as to record systemic adverse events (AEs) throughout the study. Adverse experiences were graded and recorded according to National Cancer Institute Common Toxicity Criteria for Adverse Events (NCI-CTCAE v3.0). In both studies, a dose-limiting toxicity (DLT) was defined as vaccine- or EP-related AEs including any of the following: grade 4 neutropenia; grade 3 neutropenia with fever (>38.5°C); grade 4 thrombocytopenia (≤25 × 10^9^/L); any grade 3 or 4 non-hematologic toxicity (except alopecia and inadequately treated diarrhea, nausea and vomiting); and grade 3 transaminitis (lasting ≥1 week). Grade 3 or 4 creatine phosphokinase (CPK) elevations were not considered DLTs unless they were associated with evidence of rhabdomyolysis (as assessed by renal or other organ dysfunction). Any patient experiencing a DLT was not to receive any additional vaccines, and would have automatically entered into the follow-up phase of the study. Hematology and serum chemistry laboratory safety tests were collected periodically during the study. Tests for immune dysfunction (e.g., ANA, anti-dsDNA, C3) were to be measured only if there was evidence of an autoimmune adverse experience (e.g., rash and constitutional symptoms).

For Study 1 only, patients were asked to assess the severity of the pain experienced at 1, 5, 10, and 20 minutes, and 24 hours after each V930 DNA-EP injection using 2 validated instruments: the McGill Pain Questionnaire and the Brief Pain Inventory (BPI), which included a single question (“Please rate your pain by circling the number that tells how much pain you have *right now*,” rated on a scale from 0 [no pain] to 10 [pain as bad as you can imagine]). Pain associated with the vaccination/EP procedure was assessed on the same days as vaccinations, and reported as an AE if it was considered grade 3 or 4, was a serious adverse event (SAE), or resulted in study discontinuation.

Study-related visits were divided into a vaccination period of 2 months and a follow-up period for safety and immunogenicity starting at the end of vaccinations and lasting up to 1 year. Visits 1 to 7 were done during the vaccination period, and visits 8 to13 during follow-up. During the vaccination period, physical exam and chemistry labs were performed at baseline and on days 1, 29, and 57. Evaluation of Karnofsky performance status, hematology labs, AEs, and injection sites was performed at baseline and on days 1, 15, 29, 43 and 57. Peripheral blood mononuclear cell, ELISA sample, and tumor marker collection were done at baseline and on days 1 and 43. Patients were assessed for toxicity of both the vaccine and the EP technique at each visit and at subsequent follow-up visits. Immunologic assessments for CMI were assessed at baseline and periodically during the study and follow-up.

### Laboratory assays

HER2- and CEA-specific CMI, measured by the interferon-gamma (IFN-γ) enzyme-linked immunospot (ELISPOT) assay, were determined at baseline and various times during and post vaccination, as well as ELISA for antibody responses as previously published.[33] The IFN-γ ELISPOT assay was the primary immunogenicity outcome measured and used pools of 15-mer peptides covering the entire coding sequence of the immunizing tumor antigens. In both studies, based on available data, positive immune responses were defined as both: (i) at least 35 spot forming cells per million peripheral blood mononuclear cells (35 SFC/10^6^ PBMCs), and (ii) a 3.5-fold or greater increase above background levels; this 2-dimensional criterion represents a low false positive rate of 1% or less. Staphylococcus aureus enterotoxin B (SEB) was used as a positive control in ELISPOT assays.

### Statistical considerations

In both studies, safety and tolerability were assessed by tabulating AEs and summarizing duration, intensity, and time to onset of toxicity by dose level. The incidence of vaccination-related AEs and EP-related AEs was also summarized by dose. For Study 1, summary statistics (mean, median, minimum, maximum, and standard deviation) were generated for the McGill Pain Questionnaire and BPI instrument scores at each time point measured after EP injection. HER2- and CEA-specific CMI as measured by ELISPOT were summarized at baseline and at various times during and post vaccination. If 2 or fewer CMI responses to either HER2 or CEA were observed within the 20-patient cohort receiving the 2.5-mg plasmid dose, the 90% confidence interval (CI) of the true response rate would lie completely below 30%. Likewise, the 90% CI for the true response rate would lie completely above 30% if the observed response rate for both antigens is at least 50%.

In Study 2, a Simon 2-stage optimal design was used based on CMI response [34]. In the first stage, 12 evaluable patients were to be enrolled into the high-dose V932 Ad group. If 2 or more patients had a detectable CMI response in the first stage, then 23 additional patients would be enrolled. If 6 or more of the 35 total patients enrolled were found to have a CMI response, then the design would consider the drug to warrant more extensive development. This ensured an approximate 90% chance of continuing development of the drug if the true CMI response rate was 30% and a 10% chance of continuing development if the true CMI response rate was only 10%.

## Results

### Patient demographics

A total of 33 subjects were enrolled into 2 studies from 3 centers in the United States between July 2007 and May 2009. Study 1 evaluated the safety of the V930 DNA vaccine alone. Twenty-eight patients—6 at the low dose and 22 at the high dose—were enrolled in the V930 DNA-EP alone trial (Study 1): 27 (96%) received all 5 IM V930 vaccinations followed by EP and one prematurely discontinued due to the detection of liver metastases after the fourth vaccination. Study 2 was a second phase 1 trial that evaluated the V930 DNA-EP prime-Ad boost strategy (V930 DNA-EP→V932 Ad boost study). Eleven patients were enrolled into the V930 DNA-EP→V932 Ad boost study, of which 6 eligible patients who had previously participated in Study 1. Therefore, only 5 new patients that had not previously participated in Study 1 were enrolled into Study 2. All 11 patients (5 new patients and 6 from Study 1) had received at least one injection of V930 DNA-EP: 8 (73%) received all 5 injections of V930 and at least one injection of V932 Ad; 7 (64%) received both V932 Ad injections; 6 received the low V932 Ad dose; and 5 (45%) received the high V932 Ad dose. One patient (AN333) who only received a single high dose of V932 Ad discontinued due to disease progression. Six patients (55%) completed Study 2, with 5 patients (46%) discontinuing prior to trial completion (4 due to progressive disease, while one withdrew consent).

Overall, the average patient age was approximately 60 years, and most patients were women (59%). The most common cancer diagnoses in both studies overall were colorectal cancer (36%), breast cancer (25%), and non-small cell lung cancer (21%) (Table 1). Most patients who were enrolled in both studies had advanced disease. Most patients had received prior chemotherapy with 30 (97%) having received at least one prior line of chemotherapy.

**Table 1:** Patient demographics and baseline characteristics

**Baseline characteristic**	**Study 1**		**Study 2**^**a**^	
	**V930 DNA-EP**	**V930 DNA-EP**	**V932 Ad**	**V932 Ad**
	**0.25 mg**	**2.5 mg**	**0.5 × 10**^**9 **^**vg/injection**	**0.5 × 10**^**11 **^**vg/injection**
	**(n=6)**	**(n=22)**	**(n=6)**	**(n=5)**
Age, years (mean ± SD)	66.8 ± 9.2	58.6 ± 15.9	58.8 ± 12.9	54.4 ± 8.6
Gender, n (%)				
Male	2 (33)	8 (36)	2 (33)	4 (80)
Female	4 (67)	14 (64)	4 (67)	1 (20)
Race, n (%)				
White	6 (100)	21 (96)	5 (83)	5 (100)
Other	0	1 (4.5)	1 (16.7)	0
KPS, n (%)				
100	6 (100)	17 (77)	6 (100)	5 (100)
90	0	5 (23)	0	0
Tumor diagnosis, n (%)				
Adenocarcinoma NOS	1 (17)	1 (5)	0	0
Breast cancer	0	7 (32)	3 (50)	0
Colorectal cancer	4 (67)	6 (27)	1 (17)	1 (20)
Non-small cell lung cancer	1 (17)	5 (23)	2 (33)	1 (20)
Ovarian cancer	0	1 (5)	0	0
Pancreatic cancer	0	1 (5)	0	0
Squamous cell carcinoma	0	1 (5)	0	0
NOS	0	0	0	1 (20)
Renal cancer	0	0	0	2 (40)
Bladder cancer	0	0	0	1 (20)
Prior lines of chemotherapy, n (%)				
0	0	0	0	1 (20)
1	6 (100)	11 (50)	2 (33)	1 (20)
2	0	5 (23)	3 (50)	1 (20)
≥3	0	6 (27)	1 (17)	2 (40)
Stage IV cancer	0 (0)	11 (50)	4 (67)	3 (60)

^**a**^Six eligible patients from Study 1 were enrolled into Study 2. Therefore, only 5 patients that had not previously participated in Study 1 were enrolled into Study 2. Overall 33 patients (not 39) were enrolled in both studies.

*KPS*: Karnofsky performance status; *NOS*, not otherwise specified; *SD*, standard deviation.

### Vaccine safety

The V930 DNA-EP vaccine, in the initial Phase 1 trial where DNA vaccine alone was given (Study 1), was well-tolerated, at both 0.25 mg and 2.5 mg per vaccination, with no observed DLTs. In Study 1, 71% of patients experienced a clinical (non–injection site) grade 1 or 2 AE, with fatigue (21%) being the most common. Other AEs (reported by >10% of patients) included diarrhea (25%), nausea (14%), arthralgias (14%), abdominal pain (11%), and insomnia (11%) (Table 2). The only SAE was grade 3 abdominal pain, observed in 2 patients (9%) in the 2.5-mg treatment group and considered by the investigator as definitely not drug related. No patients died during the treatment period or during the 1-year follow-up. Grade 1 or 2 AEs identified as an injection site reaction were reported in 71% of patients. The incidence of injection site AEs appeared comparable between the 0.25 mg (83%) and 2.5 mg (68%) treatment groups; none of the reported injection site AEs were worse than grade 2. The most commonly observed V930 injection site AEs included erythema (54%), site pain (46%), and swelling (32%) (Table 3).

**Table 2:** Grade 1 or 2 adverse events occurring in at least 2 patients

**Adverse event, n (%)**	**Study 1**		**Study 2**^**a**^	
	**V930 DNA-EP**^**b**^	**V930 DNA-EP**^**b**^	**V932 Ad**^**c**^	**V932 Ad**^**c**^
	**0.25 mg**	**2.5 mg**	**0.5 × 10**^**9**^	**0.5 × 10**^**11**^
			**vg/injection**	**vg/injection**
	**(n=6)**	**(n=22)**	**(n=6)**	**(n=5)**
Diarrhea	2 (33)	5 (23)	0	0
Fatigue	1 (17)	5 (23)	1 (17)	1 (20)
Arthralgias	1 (17)	3 (14)	2 (33)	0
Nausea	0	4 (18)	0	0
Skin & subcutaneous tissue disorders	1 (17)	3 (14)	1 (17)	1 (20)
Abdominal pain	0	3^a ^(14)	0	0
Infections	0	3 (14)	1 (17)	0
Insomnia	1 (17)	2 (9)	0	0
Constipation	0	2 (9)	0	0
Dizziness	0	2 (9)	1 (17)	0
Dyspnea	0	4 (18)	1 (17)	0
Hot flushes	0	2 (9)	0	0
Musculoskeletal pain	0	2 (9)	4 (67)	4^b ^(80)
Vomiting	0	2 (9)	0	0
Creatinine elevation (grade 1)	0	1 (5)	0	2 (40)

^a^Six eligible patients from Study 1 were enrolled into Study 2. Therefore, only 5 patients that had not previously participated in Study 1 were enrolled into Study 2. Overall 33 patients (not 39) were enrolled in both studies.

^b^All adverse events with DNA vaccine alone (V930 DNA-EP) were Grade 1-2, with the exception of one case of grade 3 abdominal pain felt to be unrelated to study drug in the treatment phase and one case of grade 3 abdominal pain due to small bowel obstruction in the follow-up phase believed to be related to the tumor and not related to study drug.

^c^All averse events with V930 DNA-EP→V932Ad were grades 1 or 2, with the exception of one patient who experienced grade 3 muscle spasm in the low-dose V932 Ad group and one patient with ankle pain and unilateral leg pain in the high-dose V932Ad group. Both were not related to the study drug, as determined by the investigator.

**Table 3:** Grade 1 or 2 injection site reactions (incidence ≥1% in one or more treatment groups)

**Adverse event, n (%)**	**Study 1**		**Study 2**^**a**^	
	**V930 DNA-EP**	**V930 DNA-EP**	**V932 Ad**	**V932 Ad**
	**0.25 mg**	**2.5 mg**	**0.5 × 10**^**9 **^**vg/injection**	**0.5 × 10**^**11 **^**vg/injection**
	**(n=6)**	**(n=22)**	**(n=6)**	**(n=5)**
Injection site erythema	1 (17)	14 (64)	0	3 (60)
Injection site pain	4 (67)	9 (41)	1 (17)	4 (80)
Injection site swelling	1 (17)	8 (36)	1 (17)	2 (40)
Injection site bruising	1 (17)	1(5)	0	0
Injection site papule	0	1(5)	0	0
Injection site rash	0	1(5)	0	1 (20)

^a^Six eligible patients from Study 1 were enrolled into Study 2. Therefore, only 5 patients that had not previously participated in Study 1 were enrolled into Study 2. Overall 33 patients (not 39) were enrolled in both studies.

The V932 Ad vaccine was also well-tolerated without any vaccine-related SAEs. During the combined heterologous V930 DNA-EP→V932Ad boost treatment and follow-up phase in Study 2, most patients (82%) experienced grade 1 or 2 AEs: the most common were fatigue (18%) and elevated creatinine (18%) (Table 2). Six patients (55%) had an AE identified by the investigator as study drug–related. Injection site AEs with V932 Ad appeared to be dose-related, as they were reported in a higher frequency in the higher 0.5 × 10^11^ vg/injection dose level (80%) than the lower 0.5 × 10^9^ vg/injection dose level (33%). The most commonly observed V932 Ad injection site AEs included pain (45%), erythema (27%), and swelling (27%) (Table 3). None of the reported injection site reactions were greater than grade 1. Two of 11 patients (18%) experienced grade 3 SAEs (muscle spasm and unilateral leg pain) considered not related to the vaccine. None of the 11 patients administered V932 Ad experienced a SAE during the treatment phase*.* No patient deaths were reported during the treatment phase or the 1-year follow-up period.

### Pain experienced from electroporation

It was initially anticipated that V930 DNA-EP vaccination would be tolerable, and that patients would not experience severe pain from this technique. To determine whether this indeed was the case, self-reported pain scores were collected from patients who had received V930 DNA-EP alone (Study 1). The median worst pain score reported with the McGill Pain Questionnaire, averaged over the study, was 2 (discomfort), reported by 8 of 28 patients (29%); only 4 patients (14%) rated their worst pain as either 4 (horrible) or 5 (excruciating) (Table 4). Over the entire study, 18 patients (64%) reported their worst pain as 2 or less on the 10-point Brief Pain Inventory scale, with only 2 (7%) rating their worst pain as 6 or greater.

**Table 4:** **Worst pain experienced after electroporation (Study 1) as measured by the McGill Pain Questionnaire (0-5)**^**a**^

	**V930 DNA-EP**	**V930 DNA-EP**	**Total**
	**0.25 mg**	**2.5 mg**	
	**n (%)**	**n (%)**	**n (%)**
Total number of patients	6	22	28
*Worst pain experienced by patient:*			
*entire study*^***b***^	1 (17)	3 (14)	4 (14)
0	2 (33)	4 (18)	6 (21)
1	2 (33)	6 (27	8 (29)
2	1 (17)	5 (23)	6 (21)
3	0	2 (9)	2 (7)
4	0	2 (9)	2 (7)
5			
*Worst pain experienced by patient: day 1*^***b***^			
0	1 (17)	6 (27)	7 (25)
1	3 (50)	5 (23)	8 (29)
2	2 (33)	5 (23)	7 (25)
3	0	4 (18)	4 (14)
4	0	1 (5)	1 (4)
5	0	1 (5)	1 (4)
*Worst pain experienced by patient: day 15*^***b***^			
0	3 (50)	4 (18)	7 (25)
1	3 (50)	6 (27)	9 (32)
2	0	6 (27)	6 (21)
3	0	3 (14)	3 (11)
4	0	2 (9)	2 (7)
5	0	1 (5)	1 (4)
*Worst pain experienced by patient: day 29*^***b***^			
0	3 (50)	5 (23)	8 (29)
1	1 (17)	5 (23)	6 (21)
2	1 (17)	7 (32)	8 (29)
3	1 (17)	2 (9)	3 (11)
4	0	1 (5)	1 (4)
5	0	2 (9)	2 (7)
*Worst pain experienced by patient: day 43*^***b***^			
0	2 (33)	4 (18)	6 (21)
1	2 (33)	8 (36)	10 (36)
2	2 (33)	5 (23)	7 (25)
3	0	2 (9)	2 (7)
4	0	1 (5)	1 (4)
5	0	2 (9)	2 (7)
*Worst pain experienced by patient: day 57*^***b***^			
0	2 (33)	5 (23)	7 (25)
1	2 (33)	8 (36)	10 (36)
2	1 (17)	2 (9)	3 (11)
3	1 (17)	3 (14)	4 (14)
4	0	1 (5)	1 (4)
5	0	2 (9)	2 (7)

^a^The McGill Pain Questionnaire measured pain on a 6-point scale: 0 = no pain, 1 = mild, 2 = discomforting, 3 = distressing, 4 = horrible, and 5 = excruciating.

^b^As ranked by the patient.

### Immunogenicity

**Figure 3:**
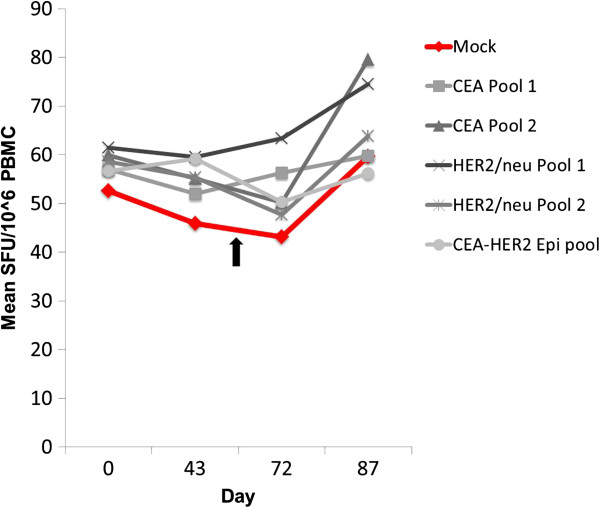
**Frequencies of CEA and HER2/neu specific IFN-γ producing T cells following high-dose V930 DNA-EP vaccination. **Longitudinal frequencies were determined from evaluable subjects (n=14); the threshold for CMI response was ≥35 SFC/10^6^ PBMC and ≥3.5-fold above mock (i.e., control well levels [red line]). Differences between time points or between CEA and HER2 and mock were not significant (*P*>0.05 by Wilcoxon rank sum test). Arrow shows day of last V930-DNA-EP vaccination.

Longitudinal antigen-specific T-cell responses to CEA, HER2, and LTB from baseline levels to approximately 4 weeks after completion of the fourth V930 vaccination (i.e., ~87 days after the first vaccination) showed that none of the 28 patients vaccinated with V930 achieved a CMI response to CEA or HER2, based on ex-vivo IFN-γ ELISPOT assays (Figure 3). None of the 11 patients vaccinated according to the heterologous V930 DNA-EP→V932 Ad approach demonstrated a measurable CMI response to CEA or HER2 (data not shown).

Subsequently, analyses designed to look for evidence of a threshold-independent CMI response were performed. These exploratory analyses were limited to 24 patients for whom ELISPOT data were available from baseline through visit 8 (2 weeks after the last of the 5 immunizations). Six of the 24 patients were in the low-dose group; the remaining 18 were in the high-dose group. Similar to the pre-specified analyses, no evidence of an increase in CEA- or HER2-specific cell mediated lymphocyte responses was observed following vaccination (*P*>0.05, paired t-test or signed-rank test) when comparing ELISPOT responses at visit 8 versus baseline (mean ELISPOT responses at visit 1 and 2).

**Figure 4:**
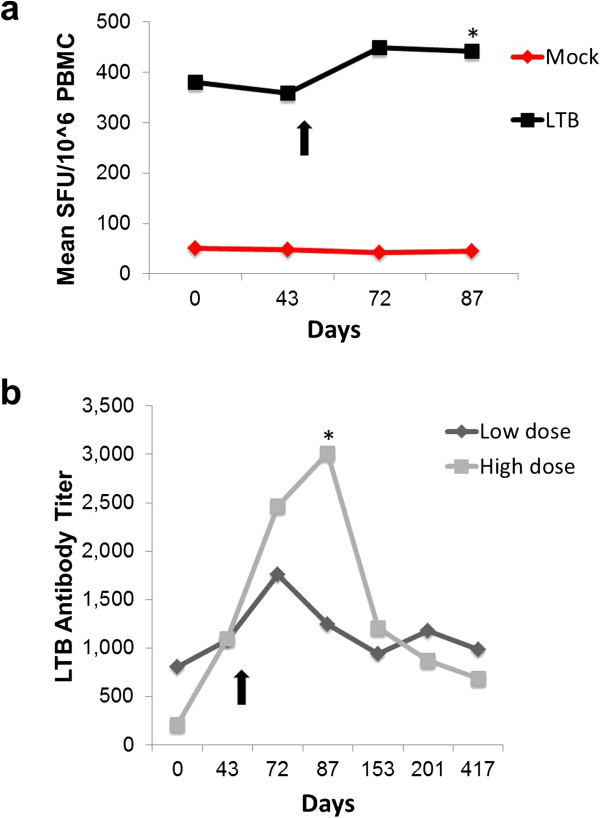
**Longitudinal cell-mediated and antibody responses to LTB. a**) Frequencies of LTB-specific IFN-γ producing T cells from evaluable subjects in both Studies 1 and 2, who had received low- or high-dose V930 DNA-EP vaccination. Arrow shows day of last V930-DNA-EP vaccination. Differences in peak values (day 87) from baseline levels were statistically significant for anti-LTB antibody responses (*P=0.03 by Wilcoxon rank sum test). **b**) Anti-LTB antibody responses. Differences in peak values (day 87) from baseline levels were statistically significant for the high-dose cohort (*P*<0.007 by Wilcoxon rank sum test). Peak levels (day 72) for the low-dose cohort were not significantly different from baseline levels (*P*>0.05).

By contrast, the LTB component of V930 DNA-EP elicited significant increases in CMI responses at day 72 post vaccination versus baseline (*P*<0.001, paired t-test or signed-rank test) (Figure 4). Based on the positive LTB ELISPOT data, antibody responses against LTB at visit 2 and 8 were determined and compared. Immunized patients had significantly higher anti-LTB antibody titers at visit 8 versus visit 2 based on anti-LTB ELISA (*P*<0.001, paired t-test or signed-rank test) (data not shown). Because of evidence of pre-existing LTB-specific T-cell and antibody responses at baseline, the immunogenicity of the LTB component of V930 could, in part, be due to recall responses to this microbial antigen. Notably, there was a trend toward a higher boosting effect in the high-dose group compared with the low-dose group.

**Figure 5:**
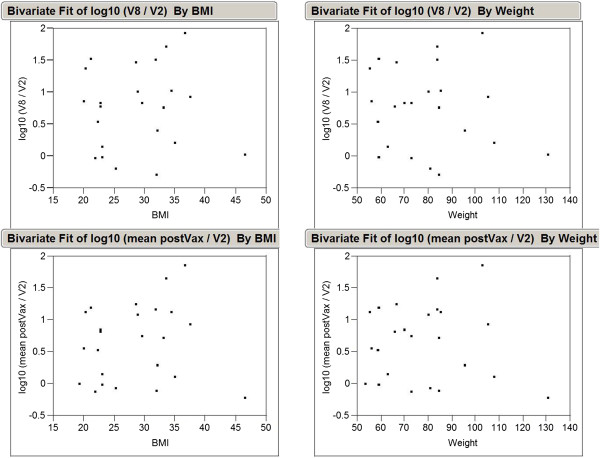
**Correlation between anti-LTB antibody response, and BMI or weight. **A bivariate analysis was performed to determine if BMI or weight were inversely correlated with an LTB response. No effect of weight or BMI was seen on the ratio of post-vaccination to pre-vaccination anti-LTB titers (*P*>0.20 in each case). LTB, *Escherichia coli *heat labile enterotoxin, B subunit; BMI, body mass index.

Due to the relatively short length of the needle used for injection of the DNA and electrodes used with the EP device, there was concern that in patients with higher body mass index (BMI), a true IM injection may not be achieved; therefore, BMI may have led to greater variability in immune responses. In order to determine whether BMI may have affected vaccination, we analyzed the relationship between weight and BMI and anti-LTB antibody titers. No significant correlation between anti-LTB antibody responses and patient weight or BMI was observed (Figure 5), suggesting that patients with higher BMIs were adequately vaccinated.

## Discussion

In this report, we describe the results of 2 phase 1 trials aimed at evaluating the safety/tolerability and immunogenicity of the bivalent DNA plasmid vaccine V930 with EP injection alone or in a heterologous approach consisting of V930 DNA-EP followed by V932 Ad boost. Similar to other previously published DNA vaccine trials,[35] our immunization regimens appeared to be safe and well-tolerated. With regards to immunogenicity, only responses to the bacterial portion of the vector were detected—none of the vaccinated subjects had detectable cell-mediated responses by ex-vivo ELISPOT to either CEA or HER2. Detectable immune responses against the LTB component of the vaccine imply that the vector was successfully delivered for antigen presentation. On the other hand, undetectable immune responses against the tumor antigens included in the vaccine supports tumor-associated antigens as poor immunogens. Even the heterologous approach, which has been reported before as enhancing immune responses, did not seem to improve the overall immunogenicity of either CEA or HER2 cancer antigens.

Weak immunogenicity of tumor antigens is perhaps the biggest challenge for cancer vaccine development, but also represents a significant opportunity for genetic vaccines [36]. DNA vaccine vectors can be readily modified to enhance gene expression, antigens can be tailored to facilitate uptake and presentation, and immunomodulatory components (e.g., danger signals or activating cytokines) can be incorporated. All these factors combined should enhance protective antitumor responses. However, encouraging results from preclinical testing of new and improved vaccine vectors are dampened by the overwhelming difficulty associated with clinical testing. Our ability to detect immune responses against the bacterial portion of the vaccine vector further validates EP as a viable option for vaccine delivery, especially those targeting pathogens. Given the intrinsic differences between immune responses against self and foreign antigens, perhaps longer availability of vaccine components (i.e., antigen and adjuvant) at the site of injection would increase the opportunity for professional antigen-presenting cells to uptake the antigen under optimal stimulatory conditions that would overcome in some extent the lack of robust immunogenicity.

A general established limitation of DNA vaccines is the injection dose of DNA. It is now understood that the clinical success of DNA vaccines in mice was greatly due to local damage caused by the hydrostatic pressure of a volume of 50 μl. Unfortunately, scaling up to an equivalent volume and dose of DNA in human subjects is not feasible with current technology; therefore, alternative methods are required. Recent data generated also point to the potential interference of 2 vectors/expression cassettes as another limitation (personal communication, G. Ciliberto & L. Aurisicchio). V930 is a mixture of 2 plasmids with the same regulatory elements (human CMV IE). Similarly, V932 Ad is a dicistronic vector where human CMV IE and mouse CMV IE drive the expression of CEA-LTB and HER2, respectively. Competition first for muscle fiber transduction, and then for transcription factors within the nucleus, may affect antigen expression levels and their immunogenicity. This may be particularly relevant for the immune response against self-antigens.

A major limitation of these 2 Phase 1 trials was that they included a rather heterogeneous population of patients (i.e., different clinical stages and cancer diagnoses). While CEA and HER2/neu are expressed in a wide variety of solid tumors, having such heterogeneity likely prevented any meaningful conclusions. Moreover, one potential explanation for the poor immunogenicity observed with the heterologous prime boost strategy with CEA and HER2 tumor antigens may be due to a large proportion of enrolled patients with metastatic disease. Because of the myriad strategies tumors employ to evade the immune system (e.g., myeloid derived suppressor cells and regulatory T-cells), a vaccine that elicits a potential immune response in the adjuvant setting may be erroneously discounted if tested in the metastatic setting as not being immunogenic enough to generate clinical activity [37,38]. The optimal setting to test vaccines in cancer patients would likely be in patients who have completed definitive curative first-line therapy, and have a high risk of recurrent disease. To our knowledge, sipuleucel-T is the only cancer vaccine strategy that has been shown in late-phase trials to have modest clinical efficacy in patients with widespread metastatic disease [1,39,40]. Interestingly, a measurable immune response against the cancer antigen prostatic acid phosphatase was detectable only in less than 30% of sipuleucel-T vaccinated patients [41]. Therefore, the sensitivity of conventional immunological assays may probably be inadequate for detection of immune responses against cancer antigens. However, a large number of other vaccine-based strategies have not been successful in the metastatic setting [42]. It is also important to note that, while the immune system in a metastatic host may block development of robust immune response to cancer vaccines, this is not universally true, and in the literature there are reports of a wide array of cancer vaccines associated with detectable immune responses in several early phase studies in patients with different metastatic solid tumors [43-48].

## Conclusions

Based on prior studies and results from our studies, one could conclude that the heterologous prime boost approach was well tolerated but ineffective with regards to generating immune responses against cancer antigens. However, due to the very small number of patients (n=5) that received the high dose of V930 DNA-EP followed by high-dose V932 Ad vaccinations, no statistically significant conclusions can be drawn regarding the heterologous prime boost approach in cancer patients. Although genetic vaccines have the potential of being therapeutic options for cancer patients with clinical benefit, there is still a need for optimization of vectors, injection schedules, and delivery methods. This will only be achieved through carefully designed and conducted clinical trials that include adequate methodology to measure relevant immune responses.

## Abbreviations

Ad: Adenovirus; ADCC: Antibody-dependent cell-mediated cytotoxicity; AE: Adverse event; BMI: Body mass index; BPI: Brief Pain Inventory; cDNA: Complementary DNA; CEA: Carcinoembryonic antigen; CI: Confidence interval; CMI: Cell-mediated immunity; CMV IE: Cytomegalovirus immediate early; CPK: Creatine phosphokinase; DLT: Dose-limiting toxicity; DNA-EP: DNA electroporation; ECD: Extracellular; ELISPOT: Enzyme-linked immunospot; EP: Electroporation; GCP: Good clinical practice; HER2: Human epidermal growth factor receptor 2; IFN-γ: Interferon-gamma; IM: Intramuscular; LTB: Labile enterotoxin; NCI-CTCAE: National cancer Institute common toxicity criteria for adverse events; PBMC: Peripheral blood mononuclear cell; SAE: Serious adverse event; SFC: Spot forming cells; TM: Transmembrane.

## Competing interests

AC received fees for participation in review activities (paid to Moffitt Cancer Center) and lectures. AB and AF are Merck employees. JC and SD are Merck employees and own stock in Merck. JM received consulting fees/honorarium and payment for lectures from Genentech and Amgen; fees for participating in review activities from Daichii; and payment for the development of educational programs. NLM was a Merck employee and owns stock in Merck. GC was a Merck employee, and is the founder of Takis. CMD, LA, JCF, ES, and AJM have no competing interests to disclose.

## Authors’ contributions

CD and AJM collected or assembled data, performed or supervised analyses, interpreted the results, wrote sections of the initial draft, provided substantial suggestions/revisions/reviews of later drafts, reviewed and confirmed that relevant conflicts of interest were disclosed, prepared the rebuttal to reviewers’ comments, and made necessary revisions to manuscript. AC provided study materials and/or patients, interpreted the results, provided substantial suggestions/revisions/reviews of later drafts, and reviewed and confirmed that relevant conflicts of interest were disclosed. LA conceived, designed, or planned the study and wrote sections of the initial draft. AB provided statistical expertise, performed or supervised analyses, interpreted the results, wrote sections of the initial draft, provided substantial suggestions/revisions/reviews of later drafts, and reviewed and confirmed that relevant conflicts of interest were disclosed. JC provided statistical expertise, performed or supervised analyses, interpreted the results, provided substantial suggestions/revisions/reviews of later drafts, and reviewed and confirmed that relevant conflicts of interest were disclosed. SD collected or assembled data, performed or supervised analyses, provided substantial suggestions/revisions/reviews of later drafts, and reviewed and confirmed that relevant conflicts of interest were disclosed. AF performed or supervised analyses, interpreted the results, wrote sections of the initial draft, provided substantial suggestions/revisions/reviews of later drafts, and reviewed and confirmed that relevant conflicts of interest were disclosed. JCF interpreted the results, wrote sections of the initial draft, provided substantial suggestions/revisions/reviews of later drafts, and reviewed and confirmed that relevant conflicts of interest were disclosed. JM conceived, designed, or planned the study, interpreted the results, wrote sections of the initial draft, provided substantial suggestions/revisions/reviews of later drafts, and reviewed and confirmed that relevant conflicts of interest were disclosed. ES conceived, designed, or planned the study, wrote sections of the initial draft, and reviewed and confirmed that relevant conflicts of interest were disclosed. NLM conceived, designed, or planned the study, provided substantial suggestions/revisions/reviews of later drafts, and reviewed and confirmed that relevant conflicts of interest were disclosed. GC conceived, designed, or planned the study, interpreted the results, wrote sections of the initial draft, and provided substantial suggestions/revisions/reviews of later drafts. All authors read and approved the final manuscript.
